# Variational quantum support vector machine based on $\Gamma$ matrix expansion and variational universal-quantum-state generator

**DOI:** 10.1038/s41598-022-10677-z

**Published:** 2022-04-26

**Authors:** Motohiko Ezawa

**Affiliations:** Department of Applied Physics, University of Tokyo, Hongo 7-3-1, Tokyo, 113-8656 Japan

**Keywords:** Quantum information, Information theory and computation

## Abstract

We analyze a binary classification problem by using a support vector machine based on variational quantum-circuit model. We propose to solve a linear equation of the support vector machine by using a $\Gamma$ matrix expansion. In addition, it is shown that an arbitrary quantum state is prepared by optimizing a universal quantum circuit representing an arbitrary $U(2^N)$ based on the steepest descent method. It may be a quantum generalization of Field-Programmable-Gate Array (FPGA).

## Introduction

Quantum computation is a hottest topic in contemporary physics^1–3^. An efficient application of quantum computations is machine learning, which is called quantum machine learning^4–17^ . A support vector machine is one of the most fundamental algorithms for machine learning^18,22,23^, which classifies data into two classes by a hyperplane. A support vector machine (SVM) is a computer algorithm that learns by examples to assign labels to objects. It is a typical method to solve a binary-classification problem^18^. The optimal hyperplane is determined by an associated linear equation $F|\psi _{ \text {in}}\rangle =|\psi _{\text {out}}\rangle$, where *F* and $|\psi _{ \text {out}}\rangle$ are given. A quantum support vector machine solves this linear equation by a quantum computer^10,13,24^. Usually, the linear equation is solved by the Harrow-Hassidim-Lloyd (HHL) algorithm^25^. However, this algorithm requires many quantum gates. Thus, the HHL algorithm is hard to be executed by using a near-term quantum computer. Actually, this algorithm has experimentally been verified only for two and three qubits^26–28^. In addition, it requires a unitary operator to execute $e^{iFt}$, which is quite hard to be implemented. The Kernel based SVM implementation based on the quantum is reported^19–21^.

The number of qubits in current quantum computers is restricted. Variational quantum algorithms are appropriate for these small-qubit quantum computers, which use both quantum computers and classical computers. Various methods have been proposed such as Quantum Approximate Optimization Algorithm (QAOA)^29^, variational eigenvalue solver^30^, quantum circuit learning^31^ and quantum linear solver^32,33^. We use wave functions with variational parameters in QAOA, which are optimized by minimizing the expectation value of the Hamiltonian. A quantum circuit has variational parameters in quantum circuit learning^31^, which are optimized by minimizing a certain cost function. A quantum linear solver solves a linear equation by variational ansatz^32,33^. The simplest method of the optimization is a steepest-descent method.

In this paper, we present a variational method for a quantum support vector machine by solving an associated linear equation based on variational quantum circuit learning. We propose a method to expand the matrix *F* by the $\Gamma$ matrices, which gives simple quantum circuits. We also propose a variational method to construct an arbitrary state by using a universal quantum circuit to represent an arbitrary unitary matrix $U(2^{N})$. We prepare various internal parameters for a universal quantum circuit, which we optimize by minimizing a certain cost function. Our circuit is capable to determine the unitary transformation *U* satisfying $U|\psi _{\text {initial} }\rangle =|\psi _{\text {final}}\rangle$ with arbitrary given states $|\psi _{ \text {initial}}\rangle$ and $|\psi _{\text {final}}\rangle$. It will be a quantum generalization of field-programmable-gate array (FPGA), which may execute arbitrary outputs with arbitrary inputs.

## Results

### Support vector machine

**Figure 1:**
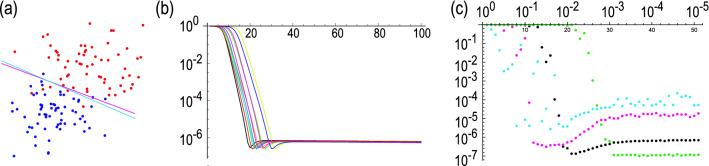
(**a**) Binary classification of red and blue points based on a quantum support vector machine with soft margin. A magenta (cyan) line obtained by an exact solution (variational method). (**b**) Evolution of the cost function. The vertical axis is the log$_{10}E_{\text {cost}}$. The horizontal axis is the variational step number. We have used $r=2$, $\xi _{1}=0.001$ and $\xi _{2}=0.0005$ and $\gamma =1$. We have runed simulations ten times, where each simulation is plotted in different color. (**c**) The saturated value of the cost function $\log _{10}E_{\text {opt}}$ as a function of $\xi _{2}$ ranging $10^{-1}\le \xi _{2}\le 10^{-5}$ for various $\xi _{1}$. The green dots indicates $\xi _{1}=0.0001$, black dots indicates $\xi _{1}=0.001$, magenta dots indicates $\xi _{1}=0.01$ and cyan dots indicates $\xi _{1}=0.1$.

A simplest example of the SVM reads as follows. Suppose that there are red and blue points whose distributions are almost separated into two dimensions. We classify these data points into two classes by a line, as illustrated in Fig. 1a.

In general, *M* data points are spattered in *D* dimensions, which we denote ${\varvec{x}}_{j}$, where $1\le j\le M$. The problem is to determine a hyperplane,

1 $$\begin{aligned} \varvec{\omega }\cdot {\varvec{x}}+\omega _{0}=0, \end{aligned}$$

separating data into two classes with the use of a support vector machine. We set

2 $$\begin{aligned} \varvec{\omega }\cdot {\varvec{x}}+\omega _{0}>0 \end{aligned}$$

for red points and

3 $$\begin{aligned} \varvec{\omega }\cdot {\varvec{x}}+\omega _{0}<0 \end{aligned}$$

for blue points. These conditions are implemented by introducing a function

4 $$\begin{aligned} f\left( {\varvec{x}}\right) =\text {sgn}\left( \varvec{\omega }\cdot {\varvec{x}}+\omega _{0}\right) , \end{aligned}$$

which assigns $f\left( {\varvec{x}}\right) =1$ to red points and $f\left( {\varvec{x}}\right) =-1$ to blue points. In order to determine $\omega _{0}$ and $\varvec{\omega }$ for a given set of data ${\varvec{x}} _{j}$, we introduce real numbers $\alpha _{j}$ by

5 $$\begin{aligned} \varvec{\omega }=\sum _{j=1}^{M}\alpha _{j}{\varvec{x}}_{j}. \end{aligned}$$

A support vector machine enables us to determine $\omega _{0}$ and $\alpha _{j}$ by solving the linear equation

6 $$\begin{aligned} F\left( \begin{array}{c} \omega _{0} \\ \alpha _{1} \\ \vdots \\ \alpha _{M} \end{array} \right) =\left( \begin{array}{c} 0 \\ y_{1} \\ \vdots \\ y_{M} \end{array} \right) , \end{aligned}$$

where $y_{i}=f(x_{i})=\pm 1$, and *F* is a $(M+1)\times (M+1)$ matrix given by

7 $$\begin{aligned} F=\left( \begin{array}{cccc} 0 &{} 1 &{} \cdots &{} 1 \\ 1 &{} &{} &{} \\ \vdots &{} &{} K+I_{M}/\gamma &{} \\ 1 &{} &{} &{} \end{array} \right) . \end{aligned}$$

Here,

8 $$\begin{aligned} K_{ij}={\varvec{x}}_{i}\cdot {\varvec{x}}_{j}, \end{aligned}$$

is a Kernel matrix, and $\gamma$ is a certain fixed constant which assures the existence of the solution of the linear equation (6) even when the red and blue points are slightly inseparable. Note that $\gamma \rightarrow \infty$ corresponds to the hard margin condition. Details of the derivation of Eq. (6) are given in Method A.

### Quantum linear solver based on $\Gamma$ matrix expansion

We solve the linear equation (6) by a quantum computer. In general, we solve a linear equation

9 $$\begin{aligned} F\left| \psi _{\text {in}}\right\rangle =c\left| \psi _{\text {out} }\right\rangle , \end{aligned}$$

for an arbitrary given non-unitary matrix *F* and an arbitrary given state $\left| \psi _{\text {out}}\right\rangle$. Here, the coefficient *c* is introduced to preserve the norm of the state, and it is given by

10 $$\begin{aligned} c=\sqrt{\left\langle \psi _{\text {in}}\right| F^{\dagger }F\left| \psi _{\text {in}}\right\rangle }. \end{aligned}$$

The HHL algorithm^25^ is a most famous algorithm to solve this linear equation by a quantum computer. We first construct a Hermitian matrix by

11 $$\begin{aligned} H=\left( \begin{array}{cc} 0 &{} F \\ F^{\dagger } &{} 0 \end{array} \right) . \end{aligned}$$

Then, a unitary matrix associated with *F* is uniquely obtained by $e^{iHt}$ . Nevertheless, it requires many quantum gates. In addition, it is a nontrivial problem to implement $e^{iHt}$.

Recently, variational methods have been proposed^32^ to solve the linear equation (9). In one of the methods, the matrix *F* is expanded in terms of some unitary matrices $U_{j}$ as

12 $$\begin{aligned} F=\sum _{j=0}^{2^{N}-1}c_{j}U_{j}. \end{aligned}$$

In general, a complicated quantum circuit is necessary to determine the coefficient $c_{j}$.

We start with a trial state $|{\tilde{\psi }}_{\text {in}}\rangle$ to determine the state $|\psi _{\text {in}}\rangle$. Application of each unitary matrix to this state is efficiently done by a quantum computer, $U_{j}|{\tilde{\psi }} _{\text {in}}\rangle =|{\tilde{\psi }}_{\text {out}}^{\left( j\right) }\rangle$, and we obtain

13 $$\begin{aligned} F|{\tilde{\psi }}_{\text {in}}\rangle =\sum _{j=0}^{2^{N}-1}c_{j}U_{j}|\tilde{\psi }_{\text {in}}\rangle =\sum _{j=0}^{2^{N}-1}c_{j}|{\tilde{\psi }}_{\text {out} }^{\left( j\right) }\rangle \equiv c|{\tilde{\psi }}_{\text {out}}\rangle , \end{aligned}$$

where $|{\tilde{\psi }}_{\text {out}}\rangle$ is an approximation of the given state $\left| \psi _{\text {out}}\right\rangle$. We tune a trial state $| {\tilde{\psi }}_{\text {in}}\rangle$ by a variational method so as to minimize the cost function^32^

14 $$\begin{aligned} E_{\text {cost}}\equiv 1-\left| \langle {\tilde{\psi }}_{\text {out}}|\psi _{ \text {out}}\rangle \right| ^{2}, \end{aligned}$$

which measures the similarity between the approximate state $|{\tilde{\psi }}_{ \text {out}}\rangle$ and the state $\left| \psi _{\text {out} }\right\rangle$ in (9). We have $0\le E_{\text {cost}}\le 1$, where $E_{\text {cost}}=0$ for the exact solution. The merit of this cost function is that the inner product is naturally calculated by a quantum computer.

Let the dimension of the matrix *F* be $2^{N}$. It is enough to use *N* satisfying $2^{N-1}<D\le 2^{N}$ without loss of generality by adding trivial $2^{N}-D$ components to the linear equation. We propose to expand the matrix *F* by the gamma matrices $\Gamma _{j}$ as

15 $$\begin{aligned} F=\sum _{j=0}^{2^{N}-1}c_{j}\Gamma _{j}, \end{aligned}$$

with

16 $$\begin{aligned} \Gamma _{j}=\bigotimes _{\beta =1}^{N}\sigma _{\alpha }^{\left( \beta \right) }, \end{aligned}$$

where $$\alpha $$ $$=0,x,y$$ and *z*.

The merit of our method is that it is straightforward to determine $c_{j}$ by the well-known formula

17 $$\begin{aligned} c_{j}=\text {Tr}\left[ \Gamma _{j}F\right] . \end{aligned}$$

In order to construct a quantum circuit to calculate $c_{j}$, we express the matrix *F* by column vectors as

18 $$\begin{aligned} F=\left\{ \left| f_{0}\right\rangle ,\cdots ,\left| f_{2^{N}-1}\right\rangle \right\} . \end{aligned}$$

We have $\left( \left| f_{q-1}\right\rangle \right) _{p}=F_{pq}$, where subscript *p* denotes the *p*-th component of $\left| f_{q-1}\right\rangle$. Then $c_{j}$ is given by

19 $$\begin{aligned} c_{j}=\sum _{q=0}^{2^{N}-1}\left( \Gamma _{j}\left| f_{q}\right\rangle \right) _{q}=\sum _{q=0}^{2^{N}-1}\left\langle \!\left\langle q\right| \right. \Gamma _{j}\left| f_{q}\right\rangle , \end{aligned}$$

where the subscript *q* denotes the ($q+1$)-th component of $\Gamma _{j}\left| f_{q}\right\rangle$. We have introduced a notation $\left| q\right\rangle \!\rangle \equiv |n_{1}n_{2}\cdots n_{N}\rangle$ with $n_{i}=0,1$, where *q* is the decimal representation of the binary number $n_{1}n_{2}\cdots n_{N}$. See explicit examples for one and two qubits in Method B.

The state $\left. \left| q\right\rangle \!\right\rangle \equiv |n_{1}n_{2}\cdots n_{N}\rangle$ is generated as follows. We prepare the NOT gates $\sigma _{x}^{\left( i\right) }$ for the *i*-th qubit if $n_{i}=1$. Using all these NOT gates we define

20 $$\begin{aligned} U_{X}^{\left( q\right) }=\bigotimes \limits _{n_{i}=1}\sigma _{x}^{\left( i\right) }. \end{aligned}$$

We act it on the initial state $\left| 0\right\rangle \!\rangle$ and obtain

21 $$\begin{aligned} U_{X}^{\left( q\right) }\left| 0\right\rangle \!\rangle =\left| q\right\rangle \!\rangle . \end{aligned}$$

Next, we construct a unitary gate $U_{f_{q}}$ generating $\left| f_{q}\right\rangle$,

22 $$\begin{aligned} U_{f_{q}}\left| 0\right\rangle \!\rangle =\left| f_{q}\right\rangle . \end{aligned}$$

**Figure 2:**
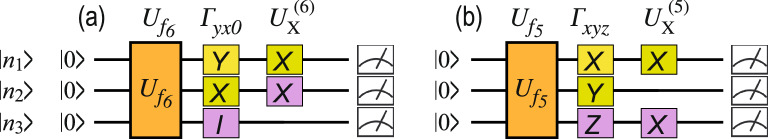
Quantum circuits determining $c_{j}$. We show an example with (**a**) $\Gamma _{yx0}=\sigma _{y}\otimes \sigma _{x}\otimes \sigma _{0}$. $U_{X}^{\left( 6\right) }\left. \left| 0\right\rangle \!\right\rangle =\sigma _{x}^{\left( 1\right) }\sigma _{x}^{\left( 2\right) }\left| 000\right\rangle =\left| 110\right\rangle =\left. \left| 6\right\rangle \!\right\rangle$ and (**b**) $\Gamma _{xyz}=\sigma _{x}\otimes \sigma _{y}\otimes \sigma _{z}$. $U_{X}^{\left( 5\right) }\left| 0\right\rangle \!\rangle = \sigma _{x}^{\left( 1\right) }\sigma _{x}^{\left( 3\right) }\left| 000\right\rangle =\left| 101\right\rangle =\left. \left| 5\right\rangle \!\right\rangle$.

We will discuss how to prepare $U_{f_{q}}$ by a quantum circuit soon later; See Eq. (33). By using these operators, $c_{j}$ is expressed as

23 $$\begin{aligned} c_{j}=\sum _{q=0}^{2^{N}-1}\left\langle \!\left\langle 0\right| \right. U_{X}^{\left( q\right) }\Gamma _{j}U_{f_{q}}\left| 0\right\rangle \!\rangle , \end{aligned}$$

which can be executed by a quantum computer. We show explicit examples in Fig. 2.

Once we have $c_{j}$, the final state is obtained by applying $\Gamma _{j}$ to $|{\tilde{\psi }}_{\text {in}}\rangle$ and taking sum over *j*, which leads to

24 $$\begin{aligned} |{\tilde{\psi }}_{\text {out}}\rangle =F|{\tilde{\psi }}_{\text {in}}\rangle =\sum _{j=0}^{2^{N}-1}c_{j}\Gamma _{j}|{\tilde{\psi }}_{\text {in}}\rangle . \end{aligned}$$

The implementation of the $\Gamma$ matrix is straightforward in quantum circuit, because the $\Gamma$ matrix is composed of the Pauli sigma matrices, as shown in Fig. 2.

### Steepest-descent method

One of the most common approaches to optimization is the steepest-descent method, where we make iterative steps in directions indicated by the gradient^34^. We may use this method to find an optimal trial state $|{\tilde{\psi }}_{\text { in}}\rangle$ closest to the state $|\psi _{\text {in}}\rangle$. To determine the gradient, we calculate the difference of the cost function $\Delta E_{\text {cost}}$ when we slightly change the trial state $|\tilde{\psi }_{\text {in}}(t)\rangle$ at step *t* by the amount of $\Delta |{\tilde{\psi }} _{\text {in}}(t)\rangle$ as

25 $$\begin{aligned} \Delta E_{\text {cost}}\equiv E_{\text {cost}}\left( |{\tilde{\psi }}_{\text {in} }(t)\rangle +\Delta |{\tilde{\psi }}_{\text {in}}(t)\rangle \right) -E_{\text { cost}}\left( |{\tilde{\psi }}_{\text {in}}(t)\rangle \right) \simeq \frac{\Delta E_{\text {cost}}}{\Delta |{\tilde{\psi }}_{\text {in}}(t)\rangle }\Delta |\tilde{ \psi }_{\text {in}}(t)\rangle . \end{aligned}$$

We explain how to construct $|{\tilde{\psi }}_{\text {in}}(t)\rangle$ by a quantum circuit soon later; See Eq. (33). Then, we renew the state as

26 $$\begin{aligned} |{\tilde{\psi }}_{\text {in}}(t)\rangle \rightarrow |{\tilde{\psi }}_{\text {in} }(t)\rangle -\eta \left( t\right) \frac{\Delta E_{\text {cost}}}{\Delta | {\tilde{\psi }}_{\text {in}}(t)\rangle }\Delta |{\tilde{\psi }}_{\text {in} }(t)\rangle , \end{aligned}$$

where we use an exponential function for $\eta _{t}$,

27 $$\begin{aligned} \eta \left( t\right) =\xi _{1}e^{-\xi _{2}t}. \end{aligned}$$

We choose appropriate constants $\xi _{1}$ and $\xi _{2}$ for an efficient search of the optimal solution, whose explicit examples are given in the caption of Fig. 1b. We stop the renewal of the variational step when the difference $\Delta |{\tilde{\psi }}_{\text {in} }(t)\rangle$ becomes sufficiently small, which gives the optimal state of the linear equation (9).

In the numerical simulation, we discretize the time step

28 $$\begin{aligned} t=n\Delta t, \end{aligned}$$

with a fixed $\Delta t$. We add a small value $\eta \left( n\Delta t\right)$ in the *p*-th component of the trial state $|{\tilde{\psi }}_{\text {in} }^{\left( p\right) }(t)\rangle$ at the *n* step

29 $$\begin{aligned} |{\tilde{\psi }}_{\text {in}}^{\left( p\right) }(\left( n+1\right) \Delta t)\rangle ={\tilde{\psi }}_{\text {in}}^{\left( p\right) }(n\Delta t)\rangle _{p}+\eta \left( t\right) \delta ^{\left( p\right) }, \end{aligned}$$

where $\delta ^{\left( p\right) }$ denotes a unit vector where only the *p* component is 1 and the other components are zero. Then, we calculate the costfunction

30 $$\begin{aligned} E_{\text {cost}}^{\left( p\right) }\left( \left( n+1\right) \Delta t\right) \equiv 1-\left| \langle {\tilde{\psi }}_{\text {in}}^{\left( p\right) }(\left( n+1\right) \Delta t)\rangle |\psi _{\text {out}}\rangle \right| ^{2}. \end{aligned}$$

By running *p* from 1 to $2^{N}$, we obtain a vector $E_{\text {cost} }^{\left( p\right) }\left( \left( n+1\right) \Delta t\right)$, whose *p*-th component is $E_{\text {cost}}^{\left( p\right) }\left( \left( n+1\right) \Delta t\right)$. Then, the gradient is numerically obtained as

31 $$\begin{aligned} \Delta E_{\text {cost}}\left( n+1\right) \equiv \left( E_{\text {cost} }^{\left( p\right) }\left( \left( n+1\right) \Delta t\right) -E_{\text {cost} }^{\left( p\right) }\left( n\Delta t\right) \right) , \end{aligned}$$

and we set the trial state at the $n+1$ step.

32 $$\begin{aligned} |{\tilde{\psi }}_{\text {in}}^{\left( p\right) }(\left( n+1\right) \Delta t)\rangle =|{\tilde{\psi }}_{\text {in}}^{\left( p\right) }(n\Delta t)\rangle +\Delta E_{\text {cost}}\left( n+1\right) . \end{aligned}$$

We iterate this process so that $\Delta E_{\text {cost}}\left( n+1\right)$ becomes sufficiently small.

We denote the saturated cost function $E_{\text {opt}}$. It depends on the choice of $\xi _{1}$ and $\xi _{2}$ in Eq. (27). We show $\log _{10}E_{ \text {opt}}$ as a function of $\xi _{2}$ for various $\xi _{1}$ in Fig. 1c. There are some features. First, $E_{\text {opt}}$ is small for small $\xi _{1}$. Namely, we need to choose small $\xi _{1}$ in order to obtain a good solution. On the other hand, the required step increases for small $\xi _{1}$. It is natural that small $\xi _{1}$ means that the step size is small. The required step number is antiproportional to $\xi _{1}$. Second, there is a critical value to obtain a good solution as a function of $\xi _{2}$ for a fixed value of $\xi _{1}$. We find that it is necessary to set $\xi _{2}<10^{-3}$.

A comment is in order. The cost function does not become zero although it becomes very small. It means that the solution is trapped by a local minimum and does not reach the exact solution. It is a general feature of variational algorithms, where we cannot obtain the exact solution. However, the exact solution is unnecessary in many cases including machine learnings. Actually, the classification shown in Fig. 1a is well done.

### Variational universal-quantum-state generator

In order to construct the trial state $|{\tilde{\psi }}_{\text {in}}(t)\rangle$, it is necessary to prepare an arbitrary state $\left| \psi \right\rangle$ by a quantum circuit. Alternatively, we need such a unitary transformation *U* that

33 $$\begin{aligned} U\left| 0\right\rangle \!\rangle =\left| \psi \right\rangle . \end{aligned}$$

It is known that any unitary transformation is done by a sequential application of the Hadamard, the $\pi /4$ phase-shift and the CNOT gates^35,36^. Indeed, an arbitrary unitary matrix is decomposable into a sequential application of quantum gates^35,36^, each of which is constructed as a universal quantum circuit systematically^37–42^. Universal quantum circuits have so far been demonstrated experimentally for two and three qubits^43–46^.

We may use a variational method to construct *U* satisfying Eq. (33). Quantum circuit learning is a variational method^31^, where angle variables $\theta _{i}$ are used as variational parameters in a quantum circuit *U*, and the cost function is optimized by tuning $\theta _{i}$. We propose to use a quantum circuit learning for a universal quantum circuit. We show that an arbitrary state $\left| \psi \left( \theta _{i}\right) \right\rangle$ can be generated by tuning $U\left( \theta _{i}\right)$ starting from the initial state $\left| 0\right\rangle \!\rangle$ as

34 $$\begin{aligned} U\left( \theta _{i}\right) \left| 0\right\rangle \!\rangle =\left| \psi \left( \theta _{i}\right) \right\rangle . \end{aligned}$$

We adjust $\theta _{i}$ by minimizing the cost function

35 $$\begin{aligned} E_{\text {cost}}\left( \theta _{i}\right) \equiv 1-\left| \left\langle \psi \left( \theta _{i}\right) \left| \psi \right\rangle \right. \right| ^{2}, \end{aligned}$$

which is the same as that of the variational quantum support vector machine. We present explicit examples of universal quantum circuits for one, two and three qubits in Method C.

**Figure 3:**
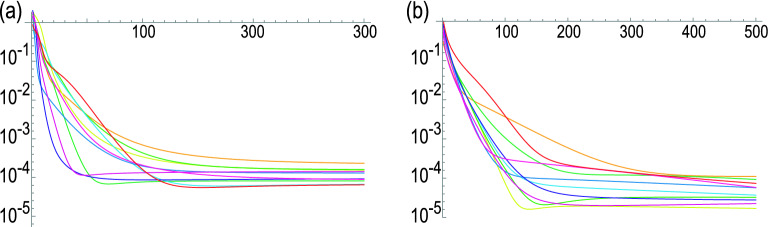
Evolution of the cost function for (**a**) two qubits and (**b**) three qubits. The vertical axis is the log$$_{10}E_{\text {cost}}$$. The horizontal axis is the number of variational steps. We use $$c_{1}=0.005$$ and $$ c_{2}=0.005$$ for both the two- and three-qubit universal quantum circuits. We prepare random initial and final states, where we have runed simulations ten times. Each simulation is plotted in different color.

### Quantum field-programmable-gate array

We next consider a problem to find a unitary transformation $U_{\text {ini-fin}}$ which maps an arbitrary initial state $\left| \psi _{\text {initial} }\right\rangle$ to an arbitrary final state $\left| \psi _{\text {final} }\right\rangle$,

36 $$\begin{aligned} U_{\text {ini-fin}}\left| \psi _{\text {initial}}\right\rangle =\left| \psi _{\text {final}}\right\rangle . \end{aligned}$$

Since we can generate an arbitrary unitary matrix as in Eq. (33), it is possible to generate such matrices $U_{\text {ini}}$ and $U_{\text {fin}}$ that

37 $$\begin{aligned} U_{\text {ini}}\left| 0\right\rangle \!\rangle =\left| \psi _{\text { initial}}\right\rangle ,\qquad U_{\text {fin}}\left| 0\right\rangle \!\rangle =\left| \psi _{\text {final}}\right\rangle . \end{aligned}$$

Then, Eq. (36) is solved as

38 $$\begin{aligned} U_{\text {fin}}=U_{\text {ini-fin}}U_{\text {ini}}, \end{aligned}$$

since $$U_{\text {ini-fin}}\left| \psi _{\text {initial}}\right\rangle =U_{ \text {ini-fin}}U_{\text {ini}}\left| 0\right\rangle \!\rangle =\left| \psi _{\text {final}}\right\rangle =U_{\text {fin}}\left| 0\right\rangle \!\rangle $$.

An FPGA is a classical integrated circuit^47–50^, which can be programmable by a customer or a designer after manufacturing in a factory. An FPGA executes any classical algorithms. On the other hand, our variational universal quantum-state generator creates an arbitrary quantum state. We program by using the variational parameters $\theta _{i}$. In this sense, the above quantum circuit may be considered as a quantum generalization of FPGA, which is a quantum FPGA (q-FPGA).

We show explicitly how the cost function is renewed for each variational step in the case of two- and three-qubit universal quantum circuits in Fig. 3, where we have generated the initial and the final states randomly. We optimize 15 parameters $\theta _{i}$ for two-qubit universal quantum circuits and 82 parameters $\theta _{i}$ for three-qubit universal quantum circuits. We find that $U_{\text {ini-fin}}$ is well determined by variational method as in Fig.3.

### Variational quantum support vector machine

We demonstrate a binary classification problem in two dimensions based on the support vector machine. We prepare a data set, where red points have a distribution around $\left( r\cos \Theta ,r\sin \Theta \right)$ with variance *r*, while blue points have a distribution around $\left( -r\cos \Theta ,-r\sin \Theta \right)$ with variance *r*. We assume the Gaussian normal distribution. We choose $\Theta$ randomly. We note that there are some overlaps between the red and blue points, which is the soft margin model.

As an example, we show the distribution of red and blue points and the lines obtained by the variational method marked in cyan and by the direct solution of (6) marked in magenta in Fig. 1a. They agrees well with one another, where both of the lines well separate red and blue points. We have prepared 31 red points and 32 blue points, and used six qubits.

## Discussion

### Efficiency

The original proposal^10^ requires $O\left( \log \left( N_{\text {D}}M\right) \right)$ runtime, where $N_{ \text {D}}$ is the dimension of the feature space and *M* is the number of training data points. It has an advantage over the classical protocol which requires $O\left( \text {polynomial}\left( N_{\text {D}},M\right) \right)$. There exisits also a quantum-inspired classical SVM^51^, which requires polynomial runtime as a function of the number of data points *M* and dimension of the feature space $N_{\text {D}}$.

*N* qubit can represent $$2^{N-1}<N_{\text {D}}M\le 2^{N}$$. Hence, the required number of qubits is $$N=\log \left( N_{\text {D}}M\right) $$. We need $$ 4^{N}-1$$ quantum gates for an exact preparation of a universal quantum state. On the other hand, a hardware-efficient universal quantum circuit prepares an approximate universal quantum state by using the order of 4*N* quantum gates^52–54^. We need *N* quantum gates for the execution of $$U_{X}^{\left( q\right) }$$ and $$\Gamma _{j}$$, separately. We need $$4^{N}+2N-1$$ quantum gates for exact preparation and 6*N* for approximate preparation. In machine learning, the exact solution is unnecessary. Thus, 6*N* quantum gates are enough.

On the other hand, the accuracy is independent of the number of required quantum gates. It is determined by $\xi _{1}$ as shown in new Fig.1c.

### Radial basis function

In this paper, we have used the linear Kernel function (8), which is efficient to classify data points linearly. However, it is not sufficient to classify data points which are not separated by the linear function. The radial basis function^55,56^ is given by

39 $$\begin{aligned} K_{ij}=\exp \left[ -\left| {\varvec{x}}_{i}-{\varvec{x}} _{j}\right| ^{2}/2\sigma ^{2}\right] , \end{aligned}$$

with a free parameter $\sigma$. It is used for a nonlinear classification^57^. It is known^58,59^ that the depth of a quantum is linear to the dimension of the feature space *N*.

## Conclusion

We have proposed that the matrix *F* is efficiently inputted into a quantum computer by using the $\Gamma$-matrix expansion method. There are many ways to use a matrix in a quantum computer such as linear regression and principal component analysis. Our method will be applicable to these cases.

Although it is possible to obtain the exact solution for the linear equation by the HHL algorithm, it requires many gates. On the other hand, it is often hard to obtain the exact solution by variational methods since trial functions may be trapped to a local minimum. However, this problem is not serious for the machine learning problem because it is more important to obtain an approximate solution efficiently rather than an exact solution by using many gates. Indeed, our optimized hyperplane also well separates red and blue points as shown in Fig. 1a.

In order to classify *M* data, we need to prepare $\log _{2}M$ qubits. It is hard to execute a large number of data points by current quantum computers. Recently, it is shown that electric circuits may simulate universal quantum gates^60–62^ based on the fact that the Kirchhoff law is rewritten in the form of the Schrödinger equation^63^. Our variational algorithm will be simulated by using them.

## Methods

### Support vector machine

**Figure 4:**
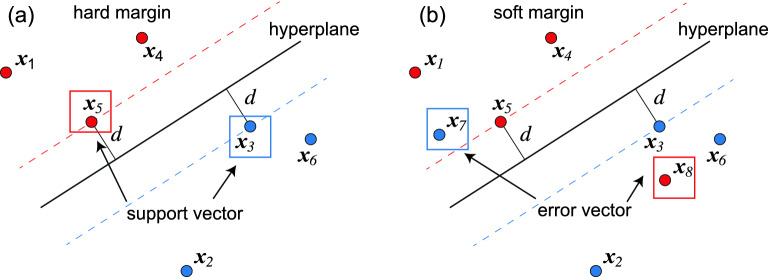
Illustration of the hyperplane and the support vector. Two support vectors are marked by red and blue squares. (**a**) Hard margin where red and blue points are separated perfectly, and (**b**) soft margin where they are separated imperfectly.

A support vector machine is an algorithm for supervised learning^18,22,23^. We first prepare a set of training data, where each point is marked either in red or blue. Then, we determine a hyperplane separating red and blue points. After learning, input data are classified into red or blue by comparing the input data with the hyperplane. The support vector machine maximizes a margin, which is a distance between the hyperplane and data points. If red and blue points are perfectly separated by the hyperplane, it is called a hard margin problem (Fig.4a). Otherwise, it is called a soft margin problem (Fig.4b).

We minimize the distance $d_{j}$ between a data point ${\varvec{x}}_{j}$ and the hyperplane given by

40 $$\begin{aligned} d_{j}=\frac{\left| \varvec{\omega }\cdot {\varvec{x}}_{j}+\omega _{0}\right| }{\left| \varvec{\omega }\right| }. \end{aligned}$$

We define support vectors ${\varvec{x}}$ as the closest points to the hyperplane. There is such a vector in each side of the hyperplane, as shown in Fig. 4a. This is the origin of the name of the support vector machine. Without loss of generality, we set

41 $$\begin{aligned} \left| \varvec{\omega }\cdot {\varvec{x}}+\omega _{0}\right| =1 \end{aligned}$$

for the support vectors, because the hyperplane is present at the equidistance of two closest data points and because it is possible to set the magnitude of $\left| \varvec{\omega }\cdot {\varvec{x}}+\omega _{0}\right|$ to be 1 by scaling $\varvec{\omega }$ and $\omega _{0}$. Then, we maximize the distance

42 $$\begin{aligned} d=\frac{\left| \varvec{\omega }\cdot {\varvec{x}}+\omega _{0}\right| }{\left| \varvec{\omega }\right| }=\frac{1}{ \left| \varvec{\omega }\right| }, \end{aligned}$$

which is identical to minimize $\left| \varvec{\omega }\right|$.

First, we consider the hard margin problem, where red and blue points are perfectly separable. All red points satisfy $$\varvec{\omega }\cdot {\varvec{x}}_{j}+\omega _{0}>1$$ and all blue points satisfy $$\varvec{ \omega }\cdot {\varvec{x}}_{j}+\omega _{0}<-1$$. We introduce variables $$ y_{j}$$, where $$y_{j}=1$$ for red points and $$y_{j}=-1$$ for blue points. Using them, the condition is rewritten as

43 $$\begin{aligned} \left( \varvec{\omega }\cdot {\varvec{x}}_{j}+\omega _{0}\right) y_{j}\ge 1 \end{aligned}$$

for each *j*. The problem is reduced to find the minimum of $\left| \varvec{\omega }\right| ^{2}$ under the above inequalities. The optimization under inequality conditions is done by the Lagrange multiplier method with the Karush-Kuhn-Tucker condition^64^. It is expressed in terms of the Lagrangian as

44 $$\begin{aligned} L\left( \varvec{\omega },\omega _{0},\varvec{\alpha }\right) =\frac{1 }{2}\left| \varvec{\omega }\right| ^{2}-\sum _{j}\beta _{j}[\left( \varvec{\omega }\cdot {\varvec{x}}_{j}+\omega _{0}\right) y_{j}-1], \end{aligned}$$

where $\beta _{j}$ are Lagrange multipliers to ensure the constraints.

For the soft margin case, we cannot separate two classes exactly. In order to treat this case, we introduce slack variables $\xi _{j}$ satisfying

45 $$\begin{aligned} \left( \varvec{\omega }\cdot {\varvec{x}}_{j}+\omega _{0}\right) y_{j}\ge 1-\xi _{j},\qquad \xi _{j}\ge 0 \end{aligned}$$

and redefine the cost function as

46 $$\begin{aligned} E_{\text {cost}}=\frac{1}{2}\left| \varvec{\omega }\right| ^{2}+\gamma \sum _{j=1}^{M}\xi _{j}^{2}. \end{aligned}$$

Here, $\gamma =\infty$ corresponds to the hard margin. The second term represents the penalty for some of data points to have crossed over the hyperplane. The Lagrangian is modified as

47 $$\begin{aligned} L\left( \varvec{\omega },\omega _{0},\xi _{i},\varvec{\beta }\right) =\frac{1}{2}\left| \varvec{\omega }\right| ^{2}+\gamma \sum _{j=1}^{M}\xi _{j}^{2}-\sum _{j=1}^{M}\left[ \left( \varvec{\omega } \cdot {\varvec{x}}_{j} +\omega _{0}\right) \beta _{j}y_{j}-\left( 1-\xi _{i}\right) \right] . \end{aligned}$$

The stationary points are determined by

48 $$\begin{aligned} \frac{\partial L}{\partial \varvec{\omega }} =&\varvec{\omega } -\sum _{j=1}^{M}\beta _{j}y_{j}{\varvec{x}}_{j}=0, \end{aligned}$$

49 $$\begin{aligned} \frac{\partial L}{\partial \omega _{0}} =&-\sum _{j=1}^{M}\beta _{j}y_{j}=0, \end{aligned}$$

50 $$\begin{aligned} \frac{\partial L}{\partial \xi _{j}} =&\gamma \xi _{j}-\beta _{j}=0, \end{aligned}$$

51 $$\begin{aligned} \frac{\partial L}{\partial \beta _{j}} =&\left( \varvec{\omega }\cdot {\varvec{x}}_{j}+\omega _{0}\right) y_{j}-\left( 1-\xi _{i}\right) =0. \end{aligned}$$

We may solve these equations to determine $\varvec{\omega }$ and $\nu _{j}$ as

52 $$\begin{aligned} \varvec{\omega }=\sum _{j=1}^{M}\beta _{j}y_{j}{\varvec{x}}_{j}, \end{aligned}$$

from (48), and

53 $$\begin{aligned} \xi _{j}=\beta _{j}/\gamma \end{aligned}$$

from (50). Inserting them into (51), we find

54 $$\begin{aligned} y_{j}\sum _{i=1}^{M}\left( \beta _{i}y_{i}{\varvec{x}}_{i}\cdot \varvec{ x}_{j} +\omega _{0}\right) -\left( 1-\beta _{j}/\gamma \right) =0. \end{aligned}$$

Since $y_{j}^{2}=1$, it is rewritten as

55 $$\begin{aligned} \omega _{0}+\sum _{i=1}^{M}\left( {\varvec{x}}_{i}\cdot {\varvec{x}} _{j}+\delta _{ij}/\gamma \right) \beta _{i}y_{i}=y_{j}. \end{aligned}$$

Since $\beta _{j}$ appears always in a pair with $y_{j}$, we introduce a new variable defined by

56 $$\begin{aligned} \alpha _{j}=\beta _{j}y_{j}, \end{aligned}$$

and we define the Kernel matrix $K_{ij}$ as

57 $$\begin{aligned} K_{ij}={\varvec{x}}_{i}\cdot {\varvec{x}}_{j}. \end{aligned}$$

Then, $\omega _{0}$ and $\alpha _{j}$ are obtained by solving linear equations

58 $$\begin{aligned} \sum _{i=1}^{M}\alpha _{j} =&0, \end{aligned}$$

59 $$\begin{aligned} \omega _{0}+\sum _{i=1}^{M}\left( {\varvec{x}}_{i}\cdot {\varvec{x}} _{j}+\delta _{ij}/\gamma \right) \alpha _{i} =&y_{j}, \end{aligned}$$

which are summarized as

60 $$\begin{aligned} \left( \begin{array}{cccc} 0 &{} 1 &{} \cdots &{} 1 \\ 1 &{} &{} &{} \\ \vdots &{} &{} K+I_{M}/\gamma &{} \\ 1 &{} &{} &{} \end{array} \right) \left( \begin{array}{c} \omega _{0} \\ \alpha _{1} \\ \vdots \\ \alpha _{M} \end{array} \right) =\left( \begin{array}{c} 0 \\ y_{1} \\ \vdots \\ y_{M} \end{array} \right) , \end{aligned}$$

which is Eq. (6) in the main text. Finally, $\varvec{\omega }$ is determined by

61 $$\begin{aligned} \varvec{\omega }=\sum _{j=1}^{M}\alpha _{j}{\varvec{x}}_{j}. \end{aligned}$$

Once the hyperplane is determined, we can classify new input data into red if

62 $$\begin{aligned} \varvec{\omega }\cdot {\varvec{x}}_{j}+\omega _{0}>0 \end{aligned}$$

and blue if

63 $$\begin{aligned} \varvec{\omega }\cdot {\varvec{x}}_{j}+\omega _{0}<0. \end{aligned}$$

Thus, we obtain the hyperplane for binary classification.

### $\Gamma$ matrix expansion

We explicitly show how to calculate $c_{j}$ in (17) based on the $\Gamma$ matrix expansion for the one and two qubits.

#### One qubit

We show an explicit example of the $\Gamma$ -matrix expansion for one qubit. One qubit is represented by a $2\times 2$ matrix,

64 $$\begin{aligned} F=\left( \begin{array}{cc} F_{11} &{} F_{12} \\ F_{21} &{} F_{22} \end{array} \right) . \end{aligned}$$

The column vectors are explicitly given by

65 $$\begin{aligned} \left| f_{1}\right\rangle =&\left( \begin{array}{c} F_{11} \\ F_{21} \end{array} \right) =F_{11}\left| 0\right\rangle +F_{21}\left| 1\right\rangle ,\qquad \end{aligned}$$

66 $$\begin{aligned} \left| f_{2}\right\rangle =&\left( \begin{array}{c} F_{12} \\ F_{22} \end{array} \right) =F_{12}\left| 0\right\rangle +F_{22}\left| 1\right\rangle . \end{aligned}$$

The coefficient $c_{j}$ in (17) is calculated as

67 $$\begin{aligned} c_{j}=\text {Tr}\left[ \sigma _{j}F\right] =\langle 0|\sigma _{j}\left| f_{1}\right\rangle +\left\langle 1\right| \sigma _{j}\left| f_{2}\right\rangle =\sum _{p=0,1}\langle p|\sigma _{j}\left| f_{p}\right\rangle =\sum _{p=0,1}\langle 0|U_{X}^{\left( p\right) }\sigma _{j}U_{f_{p}}\left| 0\right\rangle . \end{aligned}$$

#### Two qubits

Next, we show an explicit example of the $\Gamma$-matrix expansion for two qubits. Two qubits are represented by a $4\times 4$ matrix,

68 $$\begin{aligned} F=\left( \begin{array}{cccc} F_{11} &{} F_{12} &{} F_{13} &{} F_{14} \\ F_{21} &{} F_{22} &{} F_{23} &{} F_{24} \\ F_{31} &{} F_{32} &{} F_{33} &{} F_{34} \\ F_{41} &{} F_{42} &{} F_{43} &{} F_{44} \end{array} \right) . \end{aligned}$$

The column vectors are explicitly given by

69 $$\begin{aligned} \left| f_{1}\right\rangle =&\left( \begin{array}{c} F_{11} \\ F_{21} \\ F_{31} \\ F_{41} \end{array} \right) =F_{11}\left| 00\right\rangle +F_{21}\left| 01\right\rangle +F_{31}\left| 10\right\rangle +F_{41}\left| 11\right\rangle , \end{aligned}$$

70 $$\begin{aligned} \left| f_{2}\right\rangle =&\left( \begin{array}{c} F_{12} \\ F_{22} \\ F_{32} \\ F_{42} \end{array} \right) =F_{12}\left| 00\right\rangle +F_{22}\left| 01\right\rangle +F_{32}\left| 10\right\rangle +F_{42}\left| 11\right\rangle , \end{aligned}$$

71 $$\begin{aligned} \left| f_{3}\right\rangle =&\left( \begin{array}{c} F_{13} \\ F_{23} \\ F_{33} \\ F_{43} \end{array} \right) =F_{13}\left| 00\right\rangle +F_{23}\left| 01\right\rangle +F_{33}\left| 10\right\rangle +F_{43}\left| 11\right\rangle , \end{aligned}$$

72 $$\begin{aligned} \left| f_{4}\right\rangle =&\left( \begin{array}{c} F_{14} \\ F_{24} \\ F_{34} \\ F_{44} \end{array} \right) =F_{14}\left| 00\right\rangle +F_{24}\left| 01\right\rangle +F_{34}\left| 10\right\rangle +F_{44}\left| 11\right\rangle . \end{aligned}$$

The coefficient $c_{j}$ in (17) is calculated as

73 $$\begin{aligned} c_{j}=\text {Tr}\left[ \Gamma _{j}F\right] =\left\langle 00\right| \Gamma _{j}\left| f_{1}\right\rangle +\left\langle 01\right| \Gamma _{j}\left| f_{2}\right\rangle +\left\langle 10\right| \Gamma _{j}\left| f_{3}\right\rangle +\left\langle 11\right| \Gamma _{j}\left| f_{4}\right\rangle =\sum _{p=0}^{3}\langle \!\left\langle p\right| \Gamma _{j}\left| f_{p}\right\rangle =\sum _{p=0}^{3}\langle \!\left\langle 0\right| U_{X}^{\left( p\right) }\Gamma _{j}U_{f_{p}}\left| 0\right\rangle \!\rangle . \end{aligned}$$

### Universal quantum circuits

Angle variables are used as variational parameters in a universal quantum circuit learning. We present examples for one, two and three qubits.

#### One-qubit universal quantum circuit

The single-qubit rotation gates are defined by

74 $$\begin{aligned} R\left( \theta ,\phi \right) =&\exp \left[ -i\theta \left( \sigma _{x}\cos \phi +\sigma _{y}\sin \phi \right) /2\right] , \end{aligned}$$

75 $$\begin{aligned} R_{z}\left( \phi _{z}\right) =&\exp \left[ -i\sigma _{z}\phi _{z}/2\right] . \end{aligned}$$

The one-qubit universal quantum circuit is constructed as

76 $$\begin{aligned} U^{\left( 1\right) }\left( \theta ,\phi ,\phi _{z}\right) =R\left( \theta ,\phi \right) R_{z}\left( \phi _{z}\right) =\left( \begin{array}{cc} e^{-i\phi _{z}/2}\cos \frac{\theta }{2} &{} -ie^{i\left( \phi _{z}/2-\phi \right) }\sin \frac{\theta }{2} \\ -ie^{-i\left( \phi _{z}/2-\phi \right) }\sin \frac{\theta }{2} &{} e^{i\phi _{z}/2}\cos \frac{\theta }{2} \end{array} \right) . \end{aligned}$$

**Figure 5:**
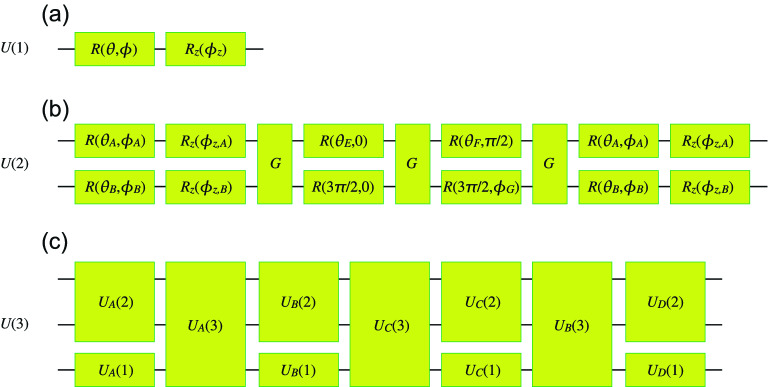
Universal quantum circuits for (**a**) one, (**b**) two and (**c**) three qubits.

We show a quantum circuit in Fig. 5a. There are three variational parameters.

It is obvious that an arbitrary state is realized starting from the state $\left| 0\right\rangle$ as

77 $$\begin{aligned} U\left( 1\right) \left( \begin{array}{c} 1 \\ 0 \end{array} \right) =\left( \begin{array}{c} e^{-i\phi _{z}/2}\cos \frac{\theta }{2} \\ -ie^{-i\left( \phi _{z}/2-\phi \right) }\sin \frac{\theta }{2} \end{array} \right) . \end{aligned}$$

#### Two-qubit universal quantum circuit

The two-qubit universal quantum circuit is constructed as^43^

78 $$\begin{aligned} U\left( 2\right) \equiv&\left[ U^{\left( 1\right) }\left( \theta _{A},\phi _{A},\phi _{z,A}\right) \otimes U^{\left( 1\right) }\left( \theta _{B},\phi _{B},\phi _{z,B}\right) \right] U_{G}\left[ R\left( \theta _{E},0\right) \otimes R\left( \frac{3\pi }{2},0\right) \right] U_{G}\left[ R\left( \theta _{F},\frac{\pi }{2}\right) \otimes R\left( \frac{3\pi }{2},\theta _{G}\right) \right] \nonumber \\&U_{G}\left[ U^{\left( 1\right) }\left( \theta _{C},\phi _{C},\phi _{z,C}\right) \otimes U^{\left( 1\right) }\left( \theta _{D},\phi _{D},\phi _{z,D}\right) \right] , \end{aligned}$$

where the entangling two-qubit gate is defined by^43^

79 $$\begin{aligned} U_{G}=e^{-i\pi /4}\exp \left[ \frac{i\pi }{4}\sigma _{z}\otimes \sigma _{z} \right] . \end{aligned}$$

The two-qubits universal quantum circuit contains 15 variational parameters. We show a quantum circuit in Fig. 5b.

#### Three-qubit universal quantum circuit

The three-qubit universal quantum circuit is constructed as

80 $$\begin{aligned} U\left( 3\right) \equiv \left[ U_{A}^{\left( 2\right) }\otimes U_{A}^{\left( 1\right) }\right] U_{A}\left( 3\right) \left[ U_{B}^{\left( 2\right) }\otimes U_{B}^{\left( 1\right) }\right] U_{C}\left( 3\right) \left[ U_{C}^{\left( 2\right) }\otimes U_{C}^{\left( 1\right) }\right] U_{B}\left( 3\right) \left[ U_{D}^{\left( 2\right) }\otimes U_{D}^{\left( 1\right) } \right] , \end{aligned}$$

where $U_{A}^{\left( 1\right) }$, $U_{B}^{\left( 1\right) }$, $U_{C}^{\left( 1\right) }$, and $U_{D}^{\left( 1\right) }$ are one-qubit universal quantum circuits, while $U_{A}^{\left( 2\right) }$, $U_{B}^{\left( 2\right) }$, $U_{C}^{\left( 2\right) }$, and $U_{D}^{\left( 2\right) }$ are two-qubit universal quantum circuit and

81 $$\begin{aligned} U_{A}\left( 3\right) =&\exp \left[ i\left( \theta _{xxz}^{A}\sigma _{x}\otimes \sigma _{x}\otimes \sigma _{z}+\theta _{yyz}^{A}\sigma _{x}\otimes \sigma _{x}\otimes \sigma _{z}+\theta _{zzz}^{A}\sigma _{z}\otimes \sigma _{z}\otimes \sigma _{z}\right) \right] , \end{aligned}$$

82 $$\begin{aligned} U_{B}\left( 3\right) =&\exp \left[ i\left( \theta _{xxz}^{B}\sigma _{x}\otimes \sigma _{x}\otimes \sigma _{z}+\theta _{yyz}^{B}\sigma _{x}\otimes \sigma _{x}\otimes \sigma _{z}+\theta _{zzz}^{B}\sigma _{z}\otimes \sigma _{z}\otimes \sigma _{z}\right) \right] , \end{aligned}$$

83 $$\begin{aligned} U_{C}\left( 3\right) =&\exp \left[ i\left( \theta _{xxx}^{C}\sigma _{x}\otimes \sigma _{x}\otimes \sigma _{x}+\theta _{yyx}^{C}\sigma _{y}\otimes \sigma _{y}\otimes \sigma _{x}+\theta _{zzx}^{C}\sigma _{z}\otimes \sigma _{z}\otimes \sigma _{x}+\theta _{00x}^{C}\sigma _{0}\otimes \sigma _{0}\otimes \sigma _{x}\right) \right] . \end{aligned}$$

Eplicit quantum circuits for $U_{A}\left( 3\right)$, $U_{B}\left( 3\right)$ and $U_{C}\left( 3\right)$ are shown in Ref.^42^. The three-qubits universal quantum circuit contains 82 variational parameters. We show a quantum circuit in Fig. 5c.

**Figure 6:**
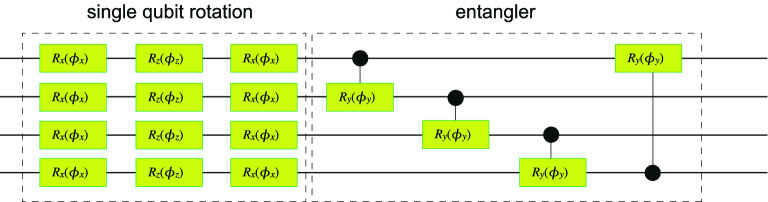
Hardware-efficient universal quantum circuits for four qubits^54^.

#### Multi-qubit universal quantum circuit

General multi-qubit universal quantum circuit is constructed in Ref.^39^. The minimum numbers of variational parameters are $4^{N}-1$ for *N*-qubit unicersal quantum circuits. However, we need more variational parameters in the currently known algorithm for $N\ge 3$.

Actually, multi-qubit universal quantum circuits are well approximated by the hardware-efficient quantum circuit^52–54^. They are constructed as

84 $$\begin{aligned} U\equiv U_{\text {CNOT}}\otimes U_{\text {Rot}}, \end{aligned}$$

with the use of the single qubit rotation

85 $$\begin{aligned} U_{\text {Rot}}\equiv \bigotimes _{n=1}^{N}\exp \left[ i\theta _{1}^{\left( n\right) }\sigma _{x}\right] \exp \left[ i\theta _{2}^{\left( n\right) }\sigma _{z}\right] \exp \left[ i\theta _{3}^{\left( n\right) }\sigma _{x} \right] , \end{aligned}$$

and the CNOT gates

86 $$\begin{aligned} U_{\text {CROT}}\equiv \bigotimes _{n=1}^{N}U_{\text {CROT}}^{n\rightarrow n+1}, \end{aligned}$$

where $U_{\text {CROT}}^{n\rightarrow n+1}$ stands for the controlled rotation gate with the controlled qubit being *n* and the target qubit being $n+1$. We need the order of 4*N* quantum gates for *N*-qubit universal quantum circuits. We show an example of the hardware-efficient quantum circuit with $N=4$ in Fig. 6.

In addition, an ansatz based on the restricted Boltzmann machine requires $$N^{2}$$ quantum gates, while a unitary-coupled cluster ansatz requires $$ N^{4}$$ quantum gates^16,34^.

### Simulations

All of the numerical calculations are carried out by Mathematica.
